# The company you keep: health behavior among work peers

**DOI:** 10.1007/s10198-019-01124-4

**Published:** 2019-10-29

**Authors:** Gerald J. Pruckner, Thomas Schober, Katrin Zocher

**Affiliations:** 1 Christian Doppler Laboratory for Aging, Health and the Labor Market, Johannes Kepler University Linz, Altenbergerstraße 69, Linz, Austria; 2 Department of Economics, Johannes Kepler University Linz, Altenbergerstraße 69, 4040 Linz, Austria

**Keywords:** Health behavior, Screening, Peer effects, Workplace, I10, I12, D83

## Abstract

There is widespread agreement that behavior crucially influences one’s health. However, little is known about what actually determines health-related behavior. We explore the impact of the place where many people spend most of their time, at work, and analyze whether an individual’s decision to participate in health screening is related to the observed behavior of peers at work. We use linked employer–employee data and exploit the transitions of workers to new jobs. We find that the health behavior of co-workers highly correlated. A comparison of individuals moving into new firms shows that participation in general health checks, mammography screening, and prostate-specific antigen tests increases with the share of work peers attending these screenings. To differentiate between peer effects and common influences at the workplace, we further separate the peer groups within firms and show that workers with similar characteristics tend to have a stronger effect on individual screening participation.

**Electronic supplementary material:**

The online version of this article (10.1007/s10198-019-01124-4) contains supplementary material, which is available to authorized users.

## Introduction

Many countries promote preventive health care because it could prolong life, increase overall well-being, and avoid costly medical treatment. Regarding the European Union, Eurostat [9] estimates that one million deaths can be prevented per year through better public health interventions. To design effective policy measures, we need to understand the motives and determinants of individual health-related behavior. In this study, we examine whether and how the work environment influences individual health behavior. In particular, we analyze to what extent general health checks and cancer screenings are related to the observed behavior of peers at work. Employees spend much time at workplaces, where social norms and information are transmitted. This transmission process may include the utilization of preventive health care measures.

A growing empirical literature explores how social interaction affects various aspects of individual behavior. Åslund and Fredriksson [2] and Markussen and Røed [21] find that welfare use and social insurance claims are contagious among neighbors and former schoolmates, in that an individual’s utilization depends on the utilization of her peers. The same effect has been found for employees’ sick leave behavior. Similar to our empirical approach, Ichino and Maggi [14] and Bradley, Green, and Leeves [4] exploit the workers moving between jobs to study the work absenteeism of teachers and bank employees. Hesselius, Nilsson, and Johansson [13], in contrast, use variation from a large-scale randomized social experiment with employees in Sweden. These studies suggest that a worker’s absenteeism level is significantly influenced by the behavior of their work colleagues. Further health behavior outcomes of friends and college roommates have been studied; for example, alcohol usage [11], obesity [6, 24, 25], and other risky health behavior measures [8]. These studies uncovered mixed results in terms of direction and significance of peer influence (see [10], for a recent review and discussion of this literature).

We find only limited empirical evidence of social interaction effects on preventive health care utilization in the literature. Findings from survey data indicate that social norms among peer groups play a role. Allen et al. [1] and Brown et al. [5], for instance, document that the perception of family and friends supporting breast cancer screening is positively related to women’s decision to participate in mammography screening. However, it remains unclear as to what extent subjective perceptions represent a causal peer effect. In contrast to these survey results, Keating et al. [16] observe the actual cancer screening behavior of peers. They use data from the Framingham Heart Study, to find that while the behavior of sisters and spouses is significantly related to women’s screening for breast and colorectal cancer, friends and co-workers have no effect. A drawback is that the data used encompass only a small number of peers who participated in the study, thus limiting the identification of peer effects.

We use comprehensive linked employer–employee data covering all private sector employees in the Austrian province of Upper Austria. These longitudinal data allow us to follow workers over time and use transition to new jobs to identify social interactions. The data can be matched with the health register data of the Upper Austrian Health Insurance Fund, which include detailed individual information on medical attendance, hospitalization, medical drug use, and participation in health screening exams. From these data, we identify the firm-level work colleagues of an individual and analyze the impact of their health behavior on the individual’s participation in general health checks and cancer screenings.

## Research design

### Empirical strategy

While it is commonly observed that peers such as friends, classmates, or work colleagues behave similarly, it is empirically challenging to identify the different channels through which the effects operate. A subject’s adaptation of behavior can depend on the peers’ behavior, attitudes, and characteristics; common opportunities; or available information.^1^ Given that many people spend the greater part of their time with colleagues at work, social and occupational surroundings can be expected to influence the health behavior of employees. We focus on workers (hereafter movers) who join a new firm and are potentially influenced in health screening behavior by their new work colleagues (hereafter, stayers).

For individual *i* who moves to firm *j* in period *t*, we estimate the following equation:

1 $$\begin{aligned} s_{ijt} = \alpha + \beta p_{jt-1} + {\varvec{X}}_{it-1} \gamma + {\varvec{F}}_{jt} \delta + \zeta s_{ijt-1} + \mu _{ijt}. \end{aligned}$$

The dependent variable $s_{ijt}$ is a binary variable indicating whether a mover participated in a medical screening examination in period *t*, that is, after he joined the new firm. In the empirical analysis, we use participation in general health checks, mammography screening, and prostate-specific antigen tests as outcome variables. To avoid potential influence of movers on stayers’ behavior [20], we measure peer behavior at a point when the subject has not yet become part of the group. In particular, $p_{jt-1}$ captures the past behavior of the new work colleagues in firm *j*. This is defined as the average past screening behavior of the stayers, that is, the number of screenings of stayers in firm *j* in period $t-1$ divided by the number of stayers $K_j$:

$$\begin{aligned} p_{jt-1}=\frac{\sum _{k=1}^{K_j} s_{kjt-1}}{K_j}. \end{aligned}$$

Given that we use the lagged behavior of stayers, the peer effect variable is exogenous with respect to the movers’ behavior and the coefficient of interest $\beta$ does not include within-firm feedback effects.

We further control for the lagged dependent variable, $s_{ijt-1}$, a set of pre-treatment individual characteristics, $X_{it-1}$, and firm-level covariates, $F_{jt}$. In the empirical analysis, period *t* covers a mover’s first two calendar years in the new firm. Period $t-1$ covers the two preceding calendar years. Stayers are defined as those who are employed in the same company for at least 4 years (i.e., in periods $t-1$ and *t*). They were already employed in firm *j* in period $t-1$ and worked for another 2 years with the movers who joined firm *j* at the beginning of period *t*.

*Identification of social interaction* For peer effects to occur, employees need to communicate about their health screening participation. These medical check-ups are preventative in nature and not associated with diseases. Taboos and social stigma of diseases may prevent employees from talking about their personal health problems.

One major concern with the approach, however, is the selection of individuals into firms due to choices of the movers themselves or firm decisions. Movers may generally collect information about the importance of primary and especially secondary health prevention in a potential new firm, including the behavior of firm employees and the existence of firm-level (health) policy measures. Firm movers might, in accordance with their own attitude toward medical prevention or health consciousness, self-select into an appropriate firm.

Conversely, the hiring firm may strongly tend to employ new staff in accordance with its corporate health and health consciousness policy for its staff. A firm attaching great importance to the healthy lifestyle of its staff to preserve their good health will tend to hire healthier employees. The companies’ procedures for recruiting this type of employees will probably put more emphasis on the applicants’ tendency toward overweight, smoking behavior, and alcohol consumption. Both employee- and firm-driven sorting entail that individuals with similar or same characteristics and attitudes would move into similar jobs.

In our empirical analysis, we control for important characteristics such as economic sector of the firm and the mover’s age, sex, past health care expenditure, and participation in previous screening exams that may affect both the choice of a firm and screening participation. The identifying assumption is that, conditional on these covariates, sorting into firms is not related to new work colleagues’ screening behavior. Job movers start working in a firm that (randomly) exposes them to peers with a higher or lower screening affinity. While in principle, we cannot rule out selection based on unobservables, we would like to argue that the most important drivers of screening participation, for example, health consciousness, are not key factors in job matching. The major determinants of an individual’s move from one firm to another are probably not those impacting the decision of whether to undergo a screening exam. A survey of employees in Austria suggests that 20% of the employees consider leaving their jobs. As the most important motives, people state their low wage (49%) and lack of career opportunities (29%) [22].

Another critical issue is that this approach cannot convincingly disentangle the peer effects from firm policy effects. A significant coefficient of our variable of interest [$p_{jt-1}$ in Eq. (1)] may reflect either the direct influence of peers on individual health behavior or shared influences at the firm level, such as health promotion measures. Some firms may undertake a persistently increased commitment to health promotion activities. Such firm policies could influence movers’ health behavior in the absence of any true peer effect.

*Peers or firm policy?* To explore this issue further, we divide the employees of a firm into groups based on their characteristics and construct different peer behavior measures. Selection mechanisms and firm policies related to health behavior should largely affect the entire workforce. In contrast, we expect that work peers with similar characteristics would exert a greater influence on an individual having the same characteristics. As regards gender, for example, we define $p_{jt-1}^f$ and $p_{jt-1}^m$ to measure the average behavior of female and male work colleagues, respectively, and estimate

2 $$\begin{aligned} s_{ijt}^{g} = \alpha + \beta _1 p_{jt-1}^f + \beta _2 p_{jt-1}^m + {\varvec{X}}_{it-1} \gamma + {\varvec{F}}_{jt} \delta + \mu _{ijt}, \end{aligned}$$

for female ($g=f$) and male ($g=m$) job movers. Similarly, we split the workforce according to job type (blue-collar vs. white-collar jobs) and age (above and below 40 years of age). We use general health check participation as our outcome variable $s_{ijt}^{g}$ in Eq. (2), given that this program targets all age groups and both sexes.

### Institutional background

Austria represents a comprehensive Bismarck-type social welfare system that includes mandatory health insurance for almost the entire population. Membership of private employees in one of the nine regional health insurance funds cannot be freely chosen, but is determined by the location of their workplace.^2^ All insured patients have access to a wide range of health care services in the inpatient and outpatient sectors. With a few exceptions, such as a small co-payment for hospitalization and prescription fees for medical drugs, health insurance covers all medical care expenses.

Insured persons above 18 years of age are entitled to the general health check program (in German, Allgemeine Vorsorgeuntersuchung). The scope and procedures of this program are regulated legally. Since its introduction in 1974, the program underwent several revisions based on developments in medical knowledge. The program offers free voluntary participation in yearly general health checks. The medical examination includes an anamnesis and a series of age- and sex-specific diagnostic and laboratory tests focusing on the identification of health risks and early detection of diseases. Following a major revision in 2005, health promotion has become an additional goal and medical doctors are asked to provide information and counseling on lifestyle choices. The questions and procedures for screening physicians were expanded and stated more precisely. Furthermore, regular invitations were sent out to increase participation of the insured.^3^

Apart from the general health check, women over 40 years of age are entitled to a mammography screening every 2 years. This screening is aimed at early detection of breast cancer using X-ray imaging. While the general health check is usually performed by a general practitioner (GP), mammography screening must be done by a radiologist.

The general health check does not by default include a prostate-specific antigen (PSA) blood test for prostate cancer. Instead, the GP provides information about the pros and cons of this test and may refer male patients to an urologist for the PSA test and further examination. In addition, men can always undergo a PSA test independent of the general health check program.

### Data and descriptives

The Austrian Social Security Database (ASSD) is a linked employer–employee dataset containing the labor market history of all private sector workers in Austria, along with individual- and firm-level characteristics [26]. We match this information with data of the regional health insurance fund for Upper Austria (in German, Oberösterreichische Gebietskrankenkasse), which include detailed information about health care utilization in the inpatient and outpatient sectors. Individual-level medical attendance data cover each single visit at the GP or medical specialist and information about participation in the general screening exam, mammography screening, and the PSA blood test, with the date of service utilization.

ASSD and health insurance data are available for the period from 1998 to 2012. First, we construct an annual panel data set of all private sector workers and their associated firms. If individuals have two jobs or move from one firm to another during a calendar year, we select the job with the higher annual earnings as their major occupation. We use this data set to identify the job movers who comprise the unit of observation in the empirical analysis. As described in “Empirical strategy”, our baseline specification allows for 2-year windows in the outcome variable, that is, we estimate an individual’s screening participation during the 2 years following the move, given that medical check-ups are typically not done annually. We additionally require that movers stay in the new firm for at least 2 years; that is, we disregard a small number of workers who switched jobs twice within two years. Additional results with 3-year time windows are presented in “Robustness checks” section.^4^

In total, we observe 181,496 persons moving to 4222 firms. Table 1 provides the descriptive statistics of the movers and stayers based on our main sample. As the table shows, 18.7% of movers and 20.7% of stayers participate in a general health screening exam in a 2-year period.^5^ The 2-year participation rates for female movers and stayers in mammography screening are 17.2% and 28.5%, respectively. The male employees’ participation rates for PSA tests are lower, at 7.9% and 14.6% for movers and stayers, respectively. The most obvious reason for the participation rate of movers being significantly lower than that of stayers is the lower age of the former group. Movers are on average 6 years younger than stayers. The lower daily wage of movers (70€ versus 80€) may also be related to age. The 2-year outpatient expenditure (medical attendance and medication) of movers and number of days they spent in hospital are significantly lower than those of the stayers, obviously because the movers are on average significantly younger. A higher percentage of movers live in urban areas (the cities of Linz, Wels, and Steyr, with a population of over 30,000 each), and, as compared to the stayers, previously worked in smaller firms.

**Table 1:** Descriptive statistics

	(1)	(2)
	Mover	Stayer
Outcome variables		
General health check	0.187	0.207
Mammography$^\mathrm{a}$	0.172	0.285
PSA test$^\mathrm{b}$	0.079	0.146
Average characteristics		
Age (years)	33.8	39.7
Female	0.419	0.401
Daily wage (€)	70	80
Outpatient expenditures (€)	694	751
Days in hospital	2.304	2.333
Urban area (Linz, Wels, Steyr)	0.178	0.119
Firm size (# employees)	549	1135
Job type		
Blue collar	0.487	0.467
White collar	0.452	0.424
*N*	181,496	602,855

This table shows the health screenings and average characteristics for movers (column (1)) and stayers (column (2)). $^{\mathrm{a,b}}$ Mammography screening refers to women, and PSA test refers to men

## Results

### Baseline estimation results

Table 2 shows the baseline specification (Eq. (1)) estimation results explaining the job movers’ attendance in general health checks, PSA tests, and mammography screening. For the general health checks in column (1), the results indicate that a person’s decision to participate is positively related to the behavior of her peers and statistically significant. An increase of 10 percentage points in the screening participation of peers increases the probability of individuals participating in health checks by 0.39 percentage points. In comparison to the movers’ average participation of 18.7%, this effect is equivalent to an increase of 2%. Similar positive and significant effects can be observed in columns (2) and (3) for prostate and breast cancer screenings, respectively. A 10 percentage point increase in participation of peers increases the PSA test participation of men by 0.22 percentage points and mammography participation of women by 0.27 percentage points.

**Table 2:** Baseline results for general health check and cancer screenings

	(1)	(2)	(3)
	General health check	PSA test	Mammography
Peer behavior	0.039*** (0.008)	0.022** (0.007)	0.027*** (0.008)
Female	0.039*** (0.002)		
Wage	0.140** (0.045)	0.330*** (0.039)	0.014 (0.064)
Lagged dependent variable	0.244*** (0.003)	0.307*** (0.007)	0.234*** (0.006)
Past healthcare utilization:			
Outpatient expenditure	0.008*** (0.001)	0.008*** (0.001)	0.015*** (0.002)
Days in hospital	$-$ 0.000 (0.000)	$-$ 0.000 (0.000)	$-$ 0.000 (0.000)
Observations	181,496	102,949	73,336
Mean of dept.	0.187	0.079	0.172

This table shows the estimation results for general health screening (column (1)), prostate cancer screening (2), and mammography screening (3). Daily wage and outpatient expenditure are measured in thousand €. Regressions additionally control for individual age, place of residence, job type, business sector, firm location, firm size, and year of job move. Standard errors clustered at the firm level are shown in parentheses, *$p<0.05$, **$p<0.01$, and ***$p<0.001$

The remaining covariates reveal the expected correlations. In line with existing empirical evidence (e.g., [15]), previous participation in the program is a strong predictor of screening uptake. For the three programs analyzed, participation during the 2 years before job move increases the probability by between 23 and 31 percentage points to participate again. The estimates for participation in general health check, in a sample of both men and women, suggest that women attend the check more often than men. A higher wage rate is positively correlated with increase in general health check and prostate cancer screening, and the age dummy coefficients^6^ reveal that general health screening increases steadily from the age of 25 up to 63. In comparison, mammography and PSA tests increase abruptly at around 40 years of age. These findings are consistent with previous results that sociodemographic factors and economic resources are important determinants of preventive healthcare decisions (e.g., [7, 17]).

Screening tests are also positively associated with overall outpatient expenditure, suggesting that people with stronger preferences or needs for outpatient medical services also invest more in preventive health care. In contrast, there is no statistically significant effect of number of days spent in hospital. Given that this variable can be interpreted as a proxy for serious health conditions, insignificant coefficients suggest that major health problems are unrelated to screening participation.

### Robustness checks

To determine the sensitivity of our results, we conducted several robustness checks with different specifications or samples. Table 3 summarizes the results. For the full estimation output of the robustness analysis, see Tables A.1–A.5 in the web appendix.

**Table 3:** Robustness checks

	General health check				Mammography				PSA test			
	(1)	(2)	(3)	(4)	(5)	(6)	(7)	(8)	(9)	(10)	(11)	(12)
	Estimate	S. e.	Mean	*N*	Estimate	S. e.	Mean	*N*	Estimate	S. e.	Mean	*N*
Baseline results	0.039***	(0.008)	0.187	181,496	0.027**	(0.008)	0.172	73,336	0.022**	(0.007)	0.079	102,949
Robustness checks												
3-Year windows	0.042***	(0.009)	0.257	120,552	0.023**	(0.008)	0.260	44,887	0.013	(0.008)	0.111	72,166
Including short-term movers	0.036***	(0.007)	0.180	209,438	0.023***	(0.007)	0.158	87,213	0.019**	(0.007)	0.076	115,828
Data since 2005	0.042***	(0.011)	0.191	115,275	0.031**	(0.009)	0.172	46,037	0.021*	(0.009)	0.080	66,031
Past 5 years’ health care	0.032**	(0.008)	0.188	168,441	0.027***	(0.008)	0.172	67,743	0.019**	(0.007)	0.079	95,865
Screening experience	0.056*	(0.022)	0.432	30,598	0.026	(0.026)	0.520	11,167	0.113*	(0.048)	0.517	6,308
Non-screener	0.035***	(0.008)	0.137	150,898	0.027***	(0.007)	0.109	62,169	0.013*	(0.007)	0.050	96,641

This table summarizes the robustness check results using different samples and specifications as indicated at the very left. Each estimate in columns (1), (5), and (9) comes from a separate regression and shows the effect of peer behavior on individual screening participation. Columns (3), (7), and (9) show the mean of the dependent variable, and columns (4), (8), and (12) show the number of observations. All regressions control for past healthcare utilization (screening participation, outpatient expenditure, days in hospital), wage, age, place of residence, job type, business sector, firm location, firm size, and year of job move. Standard errors clustered at the firm level are shown in parentheses, *$p<0.05$, **$p<0.01$, and ***$p<0.001$

For our main analysis, we use 2-year time windows for before and after job move to measure screening participation. If we instead use 3-year windows, the results would suggest a positive impact of peer behavior on participation in general health check and mammography screening. The point estimate for PSA test is also positive, but statistically insignificant ($p = 0.117$). This is most likely due to the lower sample size and participation rate in prostate cancer screening.

In our baseline analysis, we do not include movers who leave the new firm within a period of 2 years to guarantee time for social interaction. A second robustness check includes these short-term movers in the estimations. In fact, less then 15% of all movers leave the new firm early, and the coefficients using the full sample of movers are very similar compared to the baseline results. The coefficients for peer behavior decrease slightly, for example, from 0.039 to 0.036 for the general health check.

As outlined in “Institutional background”, a major expansion of the general health check program took place in 2005. This included the sending of invitation letters to insured persons. As a further robustness check, we restrict our sample to the period after 2005, assuming that program revision affected the public awareness and consequently transmission of information concerning screening programs. As can be seen from Table 3, we find very similar results in terms of effect size and statistical significance compared to our baseline results.

In our estimation approach, we use past healthcare utilization and screening behavior to allow for differences in movers’ characteristics. However, in the baseline analysis, we use the information from only 2 years before the job move, which may be insufficient to cover such differences among individuals. We, therefore, repeat the analysis, including the information on healthcare utilization for 5 years before the move. In particular, we include five dummy variables, to indicate whether the mover participated in one to five screenings, the number of days spent in hospital, and outpatient expenditure over 5 years. Given that the data are available since 1998, we use the job move data from only 2003 onward and, therefore, lose a substantial number of observations. However, from Table 3, the effect of peer behavior on individual screening participation remains statistically significant for all the three outcomes.

As a last robustness check, we differentiate between the movers with and without previous screening experience. Social interaction effects may be less relevant for individuals who participated in the past, because they are already well informed about the program. In contrast, we find statistically significant peer effects on general health check and PSA test participation for both groups. The point estimates reveal even larger effects for movers with previous screening experience. However, the average participation between these groups differs substantially, so that when compared to the means of the dependent variables, the results indicate large relative effects for non-screeners. In sum, sensitivity tests suggest that the results are robust with respect to changes in specification and sample.

### Effect heterogeneity

To study whether peer behavior has different effects depending on individual and firm characteristics, we split the sample into subsamples and estimate Eq. (1) for workers with the specified characteristics separately. We use the workers’ gender, age, job type, wage, and place of residence, as well as the number of employees in the firm, to analyze effect heterogeneity.

Table 4 summarizes the results. The general health check estimates in column (1) indicate a positive and statistically significant peer effect in all the analyzed subsamples. The point estimates suggest a larger effect on women than on men and on younger (below 40 years) than on older job movers. It is also noteworthy that because the average participation of young workers is much lower (see column (3)), the percentage effect is considerably larger for this group (2.6%). The results also show larger effects for white-collar than blue-collar jobs, and for high-wage (above median) than low-wage workers. A potential explanation is that white-collar and high-wage jobs typically entail different tasks and interactions with colleagues, which might foster peer influence.

**Table 4:** Effect heterogeneity

	General health check				Mammography				PSA test			
	(1)	(2)	(3)	(4)	(5)	(6)	(7)	(8)	(9)	(10)	(11)	(12)
	Estimate	S. e.	Mean	*N*	Estimate	S. e.	Mean	*N*	Estimate	S. e.	Mean	*N*
Baseline results	0.039***	(0.008)	0.187	181,496	0.027**	(0.008)	0.172	73,336	0.022**	(0.007)	0.079	102,949
Individual characteristics												
Men	0.032***	(0.009)	0.166	105,414								
Women	0.041***	(0.012)	0.215	76,082								
Young	0.041***	(0.009)	0.157	127,785	0.007	(0.006)	0.073	51,006	0.017*	(0.007)	0.052	93,965
Old	0.037**	(0.014)	0.258	53,711	0.064***	(0.019)	0.397	22,330	0.066	(0.039)	0.358	8,984
Blue-collar worker	0.038**	(0.013)	0.201	81,961	0.025*	(0.010)	0.171	44,069	0.011	(0.012)	0.099	35,063
White-collar worker	0.044***	(0.010)	0.179	88,325	0.029*	(0.013)	0.183	24,555	0.027**	(0.010)	0.071	61,866
Low wage	0.034***	(0.009)	0.181	90,647	0.022**	(0.008)	0.164	55,563	0.016	(0.010)	0.043	31,995
High wage	0.044***	(0.012)	0.192	90,849	0.042**	(0.015)	0.195	17,773	0.023*	(0.010)	0.095	70,954
Firm characteristics												
Small firms	0.024**	(0.009)	0.174	32,176	0.016	(0.010)	0.168	14,052	0.017	(0.011)	0.073	14,616
Large firms	0.059***	(0.015)	0.189	149,320	0.036**	(0.013)	0.173	59,284	0.028**	(0.010)	0.080	88,333

This table summarizes the effect heterogeneity in screening behavior, where each estimate in columns (1), (5), and (9) comes from a separate sample indicated at the very left. Columns (3), (7), and (9) show the mean of the dependent variable, and columns (4), (8), and (12) show the number of observations. All regressions control for past healthcare utilization (screening participation, outpatient expenditure, days in hospital), wage, age, place of residence, job type, business sector, firm location, firm size, and year of job move. Young workers are below 40 years for general health check and mammography, and old workers are beyond 40. For the PSA test, we split the sample at age 50 because the test is generally not recommended for men below that age and participation is very low below 40. Firms are defined as “small” if they have 20 employees or less, and “big” if they have more. Standard errors clustered at the firm level are shown in parentheses, *$p<0.05$, **$p<0.01$, and ***$p<0.001$

With respect to the heterogeneous results for cancer screening, the mammography (column (5)) and PSA test (column (9)) estimates for the younger and older age groups reveal a different picture compared to the results for general health checks. The point estimates suggest larger effects for older workers. However, the coefficient of the peer variable remains insignificant for the PSA test. This is most likely the consequence of the smaller sample size for the older cohort given the low number of older job movers. The peer effects with respect to cancer screening for the younger cohort are small and only weakly statistically significant. In contrast to the general health check, cancer screenings are targeted at older individuals, which may explain the heterogeneous effects. The estimation results for job type and wage reveal the same pattern as for the general health check, with larger effects for white-collar and high-wage workers than for blue-collar and low-wage workers.

The estimates of heterogeneous firm characteristics for all outcome variables in the lower part of Table 4 show stronger effects in large (more than 20 employees) than small firms. A potential explanation is the differences in firm policies with respect to firm size. Larger firms can be expected to more often introduce workplace health promotion programs, which, as outlined in “Empirical strategy”, may simultaneously affect peer and individual behavior as well as the results. The firm size may also influence the size and type of networks. Better data with information on the structure of employees within firms are necessary to address this question.

### Peer effects or firm policy?

In an attempt to disentangle the firm policy measures from peer effects, we provide additional estimation results. Table 5 summarizes the results for Eq. (2), where we define separately the measures of peer behavior according to the workers’ characteristics. In doing so, we hypothesize that peer effects mainly occur among employees if they have particular characteristics in common. For example, we can expect female workers to communicate more with other female workers and white-collar employees to have closer social contact with other white-collar workers. Similarly, younger and older cohorts may seek communication and contact in particular with employees of similar age. The estimation results suggest peer effects only among same sexes. The estimates for women moving into a new job (see column (1) of panel A) indicate a considerably stronger peer effect for females than for males, with the point estimates for only female peers statistically significant. The same holds true for male job movers, who are affected only by their male peers (see column (2)).

**Table 5:** Effect of heterogeneity in firms for general health screening

	(1)	(2)
Panel A: gender		
	Women	Men
Female peers	0.049*** (0.011)	0.004 (0.006)
Male peers	0.017 (0.010)	0.023* (0.010)
Panel B: job type		
	Blue-collar workers	White-collar workers
Blue-collar peers	0.035** (0.011)	0.007 (0.010)
White-collar peers	0.013 (0.007)	0.043** (0.013)
Panel C: age		
	Young workers	Old workers
Young peers	0.028** (0.009)	0.039** (0.014)
Old peers	0.026*** (0.007)	0.035** (0.012)

This table summarizes the effect heterogeneity in firms according to worker characteristics. Panel A shows the effect of female and male peers on women and men, panel B differentiates between blue-collar and white-collar jobs, and panel C separates the young and old workers (below and above 40 years of age). All regressions control for past healthcare utilization (screening participation, outpatient expenditure, days in hospital), wage, age, place of residence, job type, business sector, firm location, firm size, and year of job move. Standard errors clustered at the firm level are shown in parentheses, *$p<0.05$, **$p<0.01$, and ***$p<0.001$

Panel B also indicates the symmetry of peer effects for the job type of movers. We find a statistically significant effect of blue-collar peers on blue-collar workers and white-collar peers on white-collar workers, whereas the crosswise effects of the opposite groups remain insignificant. Both results indicate that it is the specific peer group that affects individual behavior, and not the firm policy or other factors shared with work colleagues.

We do not observe a similar pattern for age in panel C. The participation of both older and younger workers is correlated with peer behavior in both groups. A plausible explanation is that age is a continuous attribute making the categorization of a specific peer group difficult. Relevant work peers may often consist of individuals aged above and below the cutoff age of 40 years.

## Discussion and conclusion

In this study, we analyze whether the utilization of preventative healthcare services is related to the observed peer behavior at workplaces. When we compare the individuals moving into new firms, we find the individual participation in general heath checks, mammography screenings, and PSA tests increasing with the share of work peers attending these screenings. In addition to the causal peer mechanisms on individual participation decisions, the correlation of behavior of work peers could also be explained by the common influences at workplaces, including the firms’ hiring policy and health promotion programs. To differentiate between peer effects and common workplace impacts, we construct different peer groups within firms and provide empirical evidence that workers with similar characteristics tend to exert a larger effect on participation decisions.

The study’s findings reveal that the quantitative effects of peer behavior on individual screening participation are small. However, note that we analyze medical screening behavior within a relatively narrow time window of only 2 years after the move into a new firm. The longer a mover stays employed in a new firm, the higher the chance that work peers influence his/her screening behavior. Moreover, other (public) activities to promote preventive screening and/or increase the willingness of patients to consult a doctor also suffer from low compliance rates. Starting in 2006, Austrian health insurance funds mailed invitations for participation in general health checks to 3.5 million insured individuals belonging to predefined risk groups. Approximately 290,000 individuals accepted the invitation and underwent screening. This corresponds to a response rate of 8% [18].

A final limitation refers to the effectiveness of secondary health prevention. The more recent literature is increasingly critical of the effectiveness of certain screening measures. In particular, the PSA test has been criticized for inaccurate results and unnecessary overtreatment (e.g., [23]). If patients and/or employees are generally not convinced of the effectiveness and benefits of medical screening programs, screening participation does not adequately reflect health enhancing and promotion behavior as assumed in this study. Irrespective of this, general health checks and mammography screening are still recommended by Austrian health authorities as appropriate health promotion and preserving measures.

We conclude that work peers matter in the promotion of preventive health behavior. Channels that could explain these peer effects include the transmission of information, social norms, and beliefs with respect to health behavior and screening. If people are affected by the behavior of others at work, the workplace could act as a social multiplier of health promotion initiatives. Existing public health campaigns that directly address single individuals may be complemented by firm-level measures. Such measures could utilize the established communication channels of firms and simultaneously benefit from reinforcing peer effects.

Although this analysis is restricted to the utilization of health screening programs, similar contagion effects may exist in other behaviors such as smoking cessation, alcohol consumption, physical exercise, and nutrition. Using data on such outcomes, future research can analyze whether the results can be generalized to overall health-related behavior and explore how these social interaction effects are transmitted.

## Electronic supplementary material

Below is the link to the electronic supplementary material.

Supplementary material 1 (pdf 136 KB)

## Footnotes

1 In a seminal paper, Manski [20] distinguishes between endogenous (peer influence via group behavior), exogenous (peer influence via characteristics) and correlated (similar behavior because of similar characteristics) effects. Empirically, it is difficult to differentiate between these influences. Although we measure peer behavior in our empirical analysis, we cannot rule out that individuals are, in fact, influenced by unobserved characteristics of their peer group.

2 Separate social insurance funds offer health insurance for specific occupational groups such as farmers, civil servants, and self-employed persons.

3 See [3, § 132b] for the general goals, and Main Association of Austrian Social Security [19] and Hackl et al. [12] for details of the screening program.

4 As a further simplification, we use only the last observed job move of a person meeting these criteria to avoid multiple observations per person in the estimation data set.

5 Hackl et al. [12] report a general screening participation rate of 13 percent per year in 2010 for an Upper Austrian sample of employees and retired persons.

6 The age dummy coefficients are not shown in Table 2 but are available upon request.
