# Supplementary material for: The company you keep: health behavior among work peers
Source: Eur J Health Econ. 2019 Oct 29;21(2):251–9. doi: 10.1007/s10198-019-01124-4 (PMC7072047; doi:10.1007/s10198-019-01124-4)
Supplement: Supplementary file 1 — Supplementary material 1 (pdf 136 KB) [file 10198_2019_1124_MOESM1_ESM.pdf]

Web appendix

Table A.1: Robustness: Three year windows

	(1) General health check	(2) PSA test	(3) Mammography
Peer behavior	0.042***	0.013	0.023**
	(0.009)	(0.008)	(0.008)
Female	0.041***		
	(0.003)		
Wage	0.256***	0.488***	−0.086
	(0.065)	(0.055)	(0.087)
Lagged dependent variable	0.227***	0.290***	0.283***
	(0.004)	(0.009)	(0.007)
Past healthcare utilization:			
Outpatient expenditure	0.007***	0.005***	0.012***
	(0.001)	(0.001)	(0.002)
Days in hospital	−0.000	0.000	0.000
	(0.000)	(0.000)	(0.000)
Observations	120,552	72,166	44,887
Mean of dept.	0.257	0.111	0.260

Notes: This table shows the estimation results for participation in general health screening (column (1)), prostate cancer screening (2), and mammography screening (3). Daily wage and outpatient expenditure are measured in thousand €. Regressions additionally control for individual age, place of residence, job type, business sector, firm location, firm size, and year of job move. Standard errors clustered at the firm level are shown in parentheses,* $p < 0.05$, ** $p < 0.01$, and *** $p < 0.001$.

Table A.2: Robustness: Including short-term movers

	(1) General health check	(2) PSA test	(3) Mammography
Peer behavior	0.036***	0.019**	0.023***
	(0.007)	(0.007)	(0.007)
Female	0.036***		
	(0.002)		
Wage	0.130**	0.341***	0.031
	(0.042)	(0.036)	(0.058)
Lagged dependent variable	0.244***	0.307***	0.234***
	(0.003)	(0.007)	(0.005)
Past healthcare utilization:			
Outpatient expenditure	0.008***	0.008***	0.015***
	(0.001)	(0.001)	(0.002)
Days in hospital	−0.000	−0.000	−0.000
	(0.000)	(0.000)	(0.000)
Observations	209,438	115,828	87,213
Mean of dept.	0.180	0.076	0.158

Notes: This table shows the estimation results for general health screening (column (1)), prostate cancer screening (2), and mammography screening (3). Daily wage and outpatient expenditure are measured in thousand €. Regressions additionally control for individual age, place of residence, job type, business sector, firm location, firm size, and year of job move. Standard errors clustered at the firm level are shown in parentheses, * $p < 0.05$, ** $p < 0.01$, and *** $p < 0.001$.

Table A.3: Robustness: Data since 2005

	(1) General health check	(2) PSA test	(3) Mammography
Peer behavior	0.042***	0.021*	0.031***
	(0.011)	(0.009)	(0.009)
Female	0.040***		
	(0.003)		
Wage	0.055	0.274***	−0.088
	(0.051)	(0.046)	(0.073)
Lagged dependent variable	0.238***	0.307***	0.240***
	(0.004)	(0.008)	(0.007)
Past healthcare utilization:			
Outpatient expenditures	0.006***	0.007***	0.012***
	(0.001)	(0.001)	(0.002)
Days in hospitals	−0.000	0.000	−0.000
	(0.000)	(0.000)	(0.000)
Observations	115,275	66,031	46,037
Mean of dept.	0.191	0.080	0.172

Notes: This table shows the estimation results for participation in general health screening (column (1)), prostate cancer screening (2), and mammography screening (3). Daily wage and outpatient expenditure are measured in thousand €. Regressions additionally control for individual age, place of residence, job type, business sector, firm location, firm size, and year of job move. Standard errors clustered at the firm level are shown in parentheses,* $p < 0.05$, ** $p < 0.01$, and *** $p < 0.001$.

Table A.4: Robustness: Past 5 years healthcare utilization

	(1) General health check	(2) PSA test	(3) Mammography
Peer behavior	0.032***	0.019**	0.027***
	(0.008)	(0.007)	(0.008)
Female	0.033***		
	(0.002)		
Wage	0.057	0.245***	−0.063
	(0.044)	(0.037)	(0.061)
Number of screening participations five years before the move:			
1	0.147***	0.194***	0.184***
	(0.003)	(0.007)	(0.006)
2	0.297***	0.363***	0.354***
	(0.005)	(0.012)	(0.008)
3	0.448***	0.524***	0.466***
	(0.008)	(0.016)	(0.012)
4	0.541***	0.629***	0.550***
	(0.011)	(0.020)	(0.021)
5	0.628***	0.686***	0.628***
	(0.014)	(0.018)	(0.026)
Past healthcare utilization			
Outpatient expenditure	0.000***	0.000***	0.000***
	(0.000)	(0.000)	(0.000)
Days in hospital	0.000	−0.000	−0.000
	(0.000)	(0.000)	(0.000)
Observations	168,441	95,865	67,743
Mean of dept.	0.188	0.079	0.172

Notes: This table shows the estimation results for participation in general health screening (column (1)), prostate cancer screening (2), and mammography screening (3). Daily wage and outpatient expenditure are measured in thousand €. Regressions additionally control for individual age, place of residence, job type, business sector, firm location, firm size, and year of job move. Standard errors clustered at the firm level are shown in parentheses, * $p < 0.05$, ** $p < 0.01$, and *** $p < 0.001$.

Table A.5: Robustness: Screening experience and non-screeners

	(1) General health check experienced	(2) General health check not experienced	(3) PSA test experienced	(4) PSA test not experienced	(5) Mammography experienced	(6) Mammography not experienced
Peer behavior	0.056^{*}	0.035^{***}	0.113^{*}	0.013^{*}	0.026	0.027^{***}
	(0.022)	(0.008)	(0.048)	(0.007)	(0.026)	(0.007)
Female	0.037^{***}	0.038^{***}				
	(0.008)	(0.002)				
Wage	−0.025	0.190^{***}	0.490^{*}	0.301^{***}	0.131	−0.044
	(0.116)	(0.047)	(0.231)	(0.036)	(0.192)	(0.064)
Past healthcare utilization:						
Outpatient expenditure	0.032^{***}	0.005^{***}	0.028^{**}	0.006^{***}	0.023^{***}	0.012^{***}
	(0.005)	(0.001)	(0.009)	(0.001)	(0.005)	(0.002)
Days in hospitals	0.000	−0.000	−0.001	0.000	−0.001	0.000
	(0.000)	(0.000)	(0.001)	(0.000)	(0.001)	(0.000)
Observations	30,598	150,898	6,308	96,641	11,167	62,169
Mean of dept.	0.432	0.137	0.517	0.050	0.520	0.109

Notes: This table shows the estimation results for participation in general health screening (column (1)), prostate cancer screening (2), and mammography screening (3). Daily wage and outpatient expenditure are measured in thousand €. Regressions additionally control for individual age, place of residence, job type, business sector, firm location, firm size, and year of job move. Standard errors clustered at the firm level are shown in parentheses, * $p < 0.05$, ** $p < 0.01$, and *** $p < 0.001$.
